# Development of patatin knockdown potato tubers using RNA interference (RNAi) technology, for the production of human-therapeutic glycoproteins

**DOI:** 10.1186/1472-6750-8-36

**Published:** 2008-04-03

**Authors:** Yoon-Sik Kim, Yong-Hwa Lee, Hyun-Soon Kim, Mi-Sun Kim, Kyu-Woong Hahn, Jeong-Heon Ko, Hyouk Joung, Jae-Heung Jeon

**Affiliations:** 1 Plant Genome Research Center, KRIBB, Daejeon 305-806, Korea; 2 Department of Biological Sciences, Hannam University, Daejeon 306-791, Korea; 3 Daejeon-KRIBB-FHCRC Research Cooperation Center, KRIBB, Daejeon 305-806, Korea

## Abstract

**Background:**

Patatins encoded by a multi-gene family are one of the major storage glycoproteins in potato tubers. Potato tubers have recently emerged as bioreactors for the production of human therapeutic glycoproteins (vaccines). Increasing the yield of recombinant proteins, targeting the produced proteins to specific cellular compartments, and diminishing expensive protein purification steps are important research goals in plant biotechnology. In the present study, potato patatins were eliminated almost completely via RNA interference (RNAi) technology to develop potato tubers as a more efficient protein expression system. The gene silencing effect of patatins in the transgenic potato plants was examined at individual isoform levels.

**Results:**

Based upon the sequence similarity within the multi-gene family of patatins, a highly conserved target sequence (635 nts) of patatin gene *pat3-k1 *[GenBank accession no. DQ114421] in potato plants (*Solanum tuberosum *L.) was amplified for the construction of a patatin-specific hairpin RNAi (hpRNAi) vector. The CaMV 35S promoter-driven patatin hpRNAi vector was transformed into the potato cultivar Desiree by *Agrobacterium*-mediated transformation. Ten transgenic potato lines bearing patatin hpRNA were generated. The effects of RNA interference were characterized at both the protein and mRNA levels using 1D and 2D SDS/PAGE and quantitative real-time RT-PCR analysis. Dependent upon the patatin hpRNAi line, patatins decreased by approximately 99% at both the protein and mRNA levels. However, the phenotype (e.g. the number and size of potato tuber, average tuber weight, growth pattern, etc.) of hpRNAi lines was not distinguishable from wild-type potato plants under both *in vitro *and *ex vitro *growth conditions. During glycoprotein purification, patatin-knockdown potato tubers allowed rapid purification of other potato glycoproteins with less contamination of patatins.

**Conclusion:**

Patatin-specific hpRNAi effectively suppressed the expression of a majority of patatin variants in potato tubers via the specific degradation of individual mRNAs of the patatin multi-gene family. More importantly, patatin-knockdown potato tubers appear to be an ideal host for the production of human therapeutic glycoproteins, because they eventually allow fast, easy purification of recombinant proteins, with less contamination from potato glycoprotein patatins.

## Background

The potato (*Solanum tuberosum*) is the world's fourth most important crop. Potato tuber proteins are categorized into three groups: patatins, protease inhibitors, and other proteins [1]. Patatins are a family of glycoproteins and represent up to 40% of the total soluble protein in potato tubers [2]. Patatins accumulate in the vacuoles of tubers and leaves and are mainly found in parenchyma cells of potato tubers [3]. Two classes of patatin gene families have been identified in potato plants [4]. Class I transcripts, which lack the 22 nucleotides in the 5'-untranslated region (UTR), were found to be tuber specific, whereas class II transcripts, which bear the 22 nucleotides, were found in both tubers and roots. Patatin genes are strongly expressed in tubers but occur at very low levels in other tissues [2].

Patatins are approximately 40–45 kDa in size and are the major storage protein of potato tubers [5,6]. The roles of patatins are proposed to include fatty acid esterase, lipid acyl hydrolase, and acyltransferase activities [7-9]. Patatins are also known to have antioxidant activities [6]. However, the physiological functions of patatins in potato tubers are not fully understood.

Potato tubers have been used as a plant host for the production of recombinant human proteins and vaccines [10-13]. A study of transgenic potato plants expressing a human serum albumin gene revealed that recombinant albumin accumulated up to 0.2% of total soluble tuber protein in the plants [10]. The low level of target protein expression is a barrier to recombinant protein production in plants. In addition, the steps involved in purification of target proteins are major cost factors in plant-based protein production [12,13].

The phenomenon of gene silencing was first observed in the petunia [14]. Gene silencing is a eukaryotic genome defense system against viruses and mobile DNA elements that works by processing double-stranded RNA (dsRNA) into short interfering RNA (siRNA) [15,16]. RNA interference (RNAi) refers to a multi-step process, including the introduction of double-stranded RNA (dsRNA) into a cell, cleavage of dsRNA into short interfering RNA (siRNA) 21–26 nt in size, formation of RNA-induced silencing complex (RISC), degradation of complementary mRNA, and suppression of target gene expression [reviewed in [17,18]]. RNAi technology has become a powerful tool for the study of the functions of individual genes in a range of organisms [19-24]. Hairpin RNA (hpRNA), a class of short interfering RNA, is composed of two target sequences in an inverted repeat orientation with a spacer between. The two target sequences form a duplex RNA with a loop [21]. hpRNA interference (hpRNAi) has proven to be an effective tool for the study of the functions of several genes, including the pigment biosynthesis gene chalcone synthase (*CHS*), the ethylene-signaling gene (*EIN2*), and the flowering repression gene (*FLC1*) in Arabidopsis [21]. In this study, we utilized the hpRNAi-based reverse genetic approach to knock down various patatin isoforms in potato tubers. The objective of this study was to provide patatin knockdown potato tubers as a heterologous expression system for making human-like glycoproteins.

## Results

### Construction of a patatin-specific hairpin RNAi (hpRNAi) vector and potato transformation

**Figure 1:**
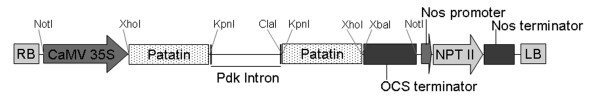
**Map of patatin-hpRNAi binary vector**. A single PCR product (nucleotides 188–798) from the target gene *pat3-k1 *was cloned into pKANNIBAL vector in an inverted-repeat orientation (sense-oriented copies in *Xho*I – *Kpn*I site; anti-sense oriented copies in *Cla*I – *Xba*I site). pKANNIBAL vector was digested with *Not*I enzyme and then *Not*I fragments were cloned into pART27 binary vector.

**Figure 2:**
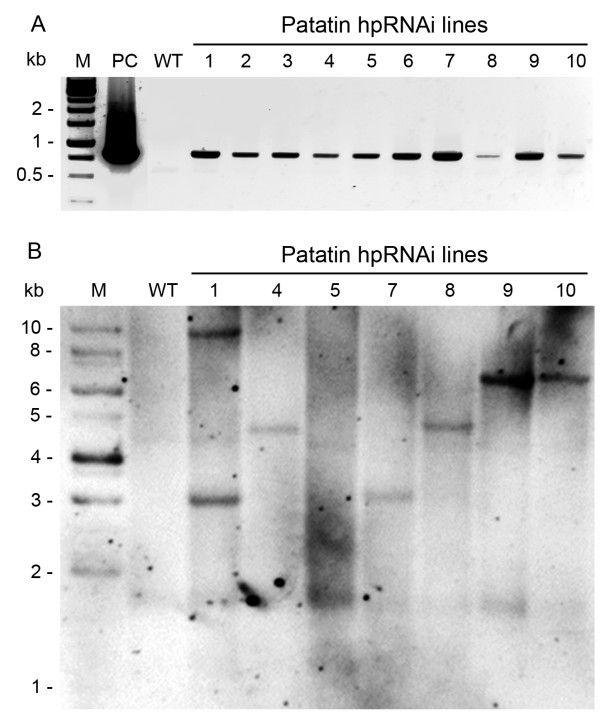
**Characterization of T-DNA insertions in patatin hpRNAi lines**. (A) PCR amplification of *npt *II gene in ten patatin hpRNAi lines. The PCR results confirmed that lanes 1 to 10 are patatin hpRNAi lines. Lane PC (DNA of hpRNAi vector was used as a template for PCR) or lane WT (genomic DNA of WT plants was used as a template for PCR) is the positive or negative control, respectively. Lanes 1 to 10 are patatin hpRNAi potato lines. (B) DNA gel blot of wild-type and seven patatin hpRNAi lines. Genomic DNA was digested with *Xba*I and probed with *npt *II gene probe. Lines 4, 7, 8, and 10 each have one T-DNA insertion.

Prior to the construction of an hpRNAi vector for suppression of patatin expression, the sequences of 29 known genes in the patatin multi-gene family in the potato were downloaded from the NCBI database and aligned for a thorough comparison of the genes. Great similarities (> 90%) between the patatin genes were found to exist at the nucleotide level (data not shown). A highly conserved 635 nt fragment of *pat3-k1 *(GenBank: DQ114421), nucleotides 188–798, was PCR amplified using Desiree as template. Half of the PCR fragments were inserted into the *Xho*I – *Kpn*I site of pKANNIBAL vector in the sense orientation and the other half of the PCR fragments were inserted into the *Xba*I – *Cla*I site in the antisense orientation. The patatin-hpRNAi frame in pKANNIBAL was confirmed by restriction analysis and nucleotide sequencing. The pKANNIBAL vector was digested with *Not*I enzyme. *Not*I fragments were cloned into pART27 binary vector, resulting in a patatin-specific hpRNAi vector, which was driven by CaMV 35S promoter (Figure 1). The patatin hpRNAi binary vector was transformed into potato plants (cv. Desiree) via *Agrobacterium*-mediated transformation. Ten independent patatin-hpRNAi potato lines were selected on kanamycin. The transgene in the hpRNAi lines was confirmed by PCR screening with *npt *II primers (Figure 2A) and CaMV 35S primers (data not shown). All ten of these lines had the *npt *II gene and were resistant to kanamycin. Seven of the regenerated kanamycin-resistant potato plants were further analyzed by Southern blot to determine the number of T-DNA inserts (Figure 2B). Patatin hpRNAi lines 4, 7, 8, and 10 each had one T-DNA insert.

### Analysis of patatins on 1D SDS-PAGE gel

**Figure 3:**
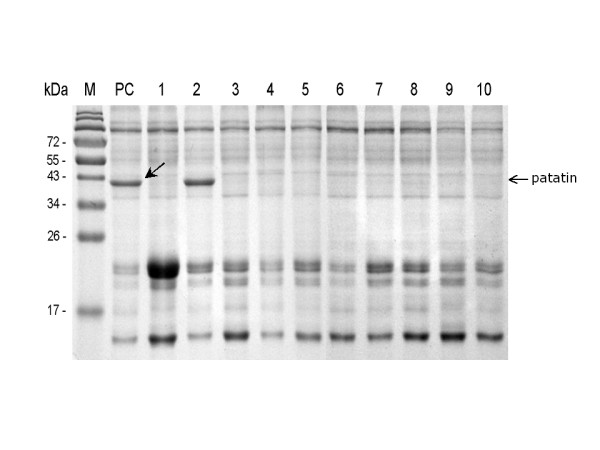
**SDS-PAGE analysis of total soluble protein of potato tuber**. Total tuber protein was extracted from mature potato tubers of (about 2 month old) the hpRNAi and WT plants grown in greenhouse. Extracts were separated on 10% SDS-PAGE gel and stained with coomassie blue. Patatin was found to be approximately 40 kDa in size (as indicated in arrow). Lane M, molecular mass marker; lane PC, wild-type positive control; lane 1–10, patatin hpRNAi lines. 10 μg of total soluble protein was loaded in each well. Marker sizes (in kDa) are indicated.

To determine the relative efficacy of gene silencing between patatin hpRNAi lines, one-dimensional (1D) SDS-PAGE analysis was initially performed using total soluble protein extracted from two month old mature potato tubers of the hpRNAi and WT plants grown in greenhouse. As expected, the total protein of WT plants showed a thick band corresponding to patatin (~40 kDa) on the SDS-PAGE gel (Figure 3). To our surprise, except for line 2, the patatin hpRNAi lines did not show patatin bands on the gel. The patatin level in hpRNAi line 2 was similar to the WT plants. Based on the amounts of patatins, gene silencing seemed to be highly effective in the individual lines where the patatin-hpRNA was expressed. These data indicate that the inverted repeat sequence of partial patatin gene *pat3-k1 *(635 nts) effectively induces gene silencing.

### Analysis of patatin variants on 2D PAGE gel

Based upon the levels of patatin in the 1D gel, we chose four patatin knockdown lines (line 4, 5, 8, and 10) for further analysis of various patatin variants using two-dimensional (2D) PAGE gel electrophoresis. Our 2D gel analysis of WT and line 4 mature tuber protein confirmed that the presumed multiple patatin spots [25] at 43 kDa had disappeared. Other patatin hpRNAi lines (lines 5, 8, and 10) showed patterns in patatin reduction similar to line 4 (See Additional file 1). Overall, 2D gel analysis of the patatin-hpRNAi line 4 revealed that the number and size of patatin spots significantly reduced, whereas other protein spots almost remained unchanged.

### Analysis of pat3-k1 mRNAs in hpRNAi lines using real-time RT-PCR

**Figure 4:**
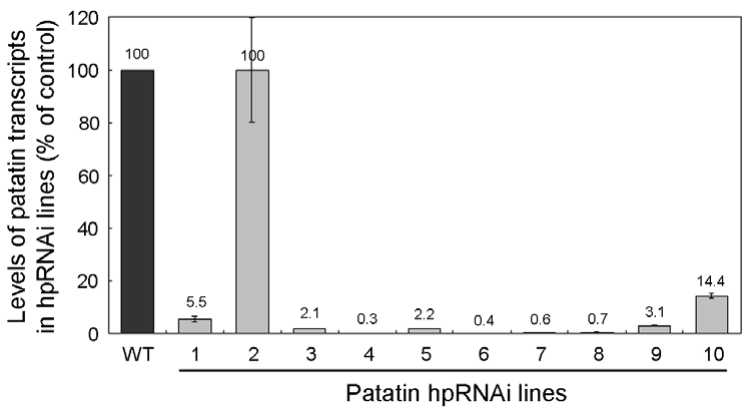
**Real-time RT-PCR of patatin hpRNAi lines**. Real-time RT-PCR was performed on RNA extracted from 10 independent hpRNAi lines using primers designed against the patatin gene (*pat3-k1*). WT is a wild-type positive control. Lanes 1 to 10 are ten independent patatin hpRNAi lines. 18S rRNA was amplified as a control for equal amounts of RNA templates. Real-time RT-PCR results show relative fold changes of *pat3-k1 *transcript levels in hpRNAi lines in comparison with WT. The error bars indicate mean ± SD. Mean and SD were derived by two independent experiments. *pat3-k1 *transcript levels were significantly reduced in most hpRNAi lines, compared to the WT control.

To confirm the data obtained from 1D SDS-PAGE and 2D PAGE gel electrophoresis, a real-time RT-PCR was performed using total RNA extracted from mature potato tubers (two month old) of ten independent patatin-hpRNAi lines grown in greenhouse. We used a primer pair designed against the most highly conserved sequence in the patatin gene family. The levels of various patatin transcripts present in hpRNAi lines were measured by a quantitative real-time RT-PCR with the primers. As shown in Figure 4, real-time RT-PCR revealed that patatin transcripts in ten patatin-hpRNAi lines were significantly reduced. Patatin hpRNAi lines 4, 5, 7, 8 and 10 showed significant reductions (95 to 99%) in patatin transcripts compared to WT plants. These data are consistent with the data obtained from 1D and 2D gel analysis. The expression of the patatin-hpRNA presumably led to effective inhibition of the expression of the *pat3-k1 *gene and other members of the gene family.

### *In vitro*- and *ex vitro*-grown patatin-hpRNAi lines showed a normal growth pattern and phenotype

To examine the effects of gene silencing on the phenotype, tuber number, and average tuber weight of patatin-hpRNAi potato plants, the transgenic potato plants were cultured *in vitro *and also grown in a greenhouse. During *in vitro *culture, the transgenic potato plants showed a normal morphologic characteristic and growth pattern in patatin hpRNAi lines 2, 4, 7, 8 (See Additional file 2A). Ten patatin-hpRNAi greenhouse-grown potato plants were harvested and the number of tubers per potato plant and average tuber weight were compared with wild-type (See Additional file 2B). The number of tubers in patatin hpRNAi lines ranged from 2.7 to 6.4, whereas that of wild-type plants was 6.8. Average tuber weight in patatin hpRNAi lines and wild-type was variable between the plants. Though average tuber weight of patatin hpRNAi lines seemed to be a bit smaller than that of wild-type, there were no significant differences (*P *< 0.01) (See Additional file 2C). Overall, patatin-knockdown potato plants showed a normal growth pattern and phenotype during *in vitro *culture and greenhouse cultivation.

### Patatin-knockdown potatoes reduced additional purification step of glycoproteins

**Figure 5:**
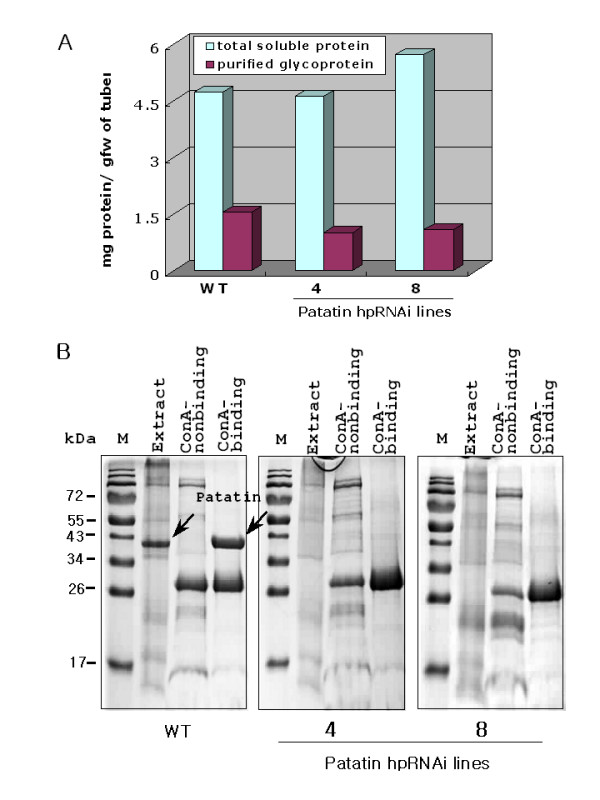
**Analysis of ConA purified glycoproteins in WT and patatin-hpRNAi lines**. (A) Determination of total soluble proteins and ConA purified glycoproteins by Bradford assay. One gram of fresh weight of tuber was used for the extraction of total soluble proteins and purification of glycoprotein. (B) SDS-PAGE analysis of ConA purified glycoproteins in WT and patatin-hpRNAi line 4 and 8. Extract (total soluble proteins), ConA-nonbinding and ConA-binding fractions were separated on 10% SDS-PAGE gel and stained with coomassie blue. Patatin was found to be approximately 40 kDa in size in wild-type plants only. Lane M, molecular mass marker. 20 μg of each fraction was loaded in each well. Marker sizes (in kDa) are indicated.

Patatin-knockdown potatoes are likely to have a few advantages in terms of glycoprotein purification, compared to wild-type potatoes. To confirm the possible advantages of patatin-knockdown potatoes, potato glycoproteins were purified by affinity chromatography on Sepharose-coupled concanavalin A (ConA) and the purified glycoproteins were monitored by running a SDS-PAGE gel (Figure 5). The content of total soluble proteins was not reduced in transgenic plants, though patatin was almost completely knocked down. In our purification system, the yields of glycoproteins were 32.6% (wild-type), 21.9% (line 4) and 19.3% (line 8) (Figure 5A). The level of glycoproteins in WT was higher than transgenic plants. In addition, SDS-PAGE analysis showed that the fraction of purified glycoproteins in wild-type potatoes has two major bands (Figure 5B). One was patatin and the other was a band in size 26–30 kDa. In contrast, patatin-RNAi lines had only one major band (26–30 kDa). Therefore, no further purification steps to remove patatin were required.

## Discussion

Patatins are considered to be a major storage protein in potato tubers [5]. Here we report that by suppressing the expression of the potato patatin multi-gene family, transgenic potato tubers accumulated dramatically low levels of patatins. This was achieved by RNA interference (RNAi) that was first discovered in *Caenorhabditis elegans *[26]. In the present study, we successfully employed RNAi technology to produce patatin knockdown/free potato tubers to eventually provide potato tubers as a heterologous expression system for the production of human therapeutic glycoproteins.

The potato genome (cv. Desiree) has 64–72 patatin DNA copies, and most of the DNA copies do not show much allelic variation based on restriction fragment length polymorphism (RFLP) mapping [27]. A study of patatins present in cv. Kuras showed that patatins are 84–96% identified at amino acid sequence levels [5]. When Kuras tuber protein spots from Coomassie Brilliant Blue-stained 2D gels were assigned using tryptic PMF analysis, the major acidic protein spots, 40–45 kDa in size, were patatin variants, and more than 15 patatin spots were observed on the 2D gel [5]. All known potato patatins are grouped into a large family of multi-genes sharing 80–95% identity at the nucleotide level and clustered into three clades including pat1, pat2, and pat3 [5]. When we aligned nucleotide sequences of 29 patatin genes from Kuras and Kennebec, patatins of both cultivars had more than 90% identity. Thus, RNAi appeared to be a suitable reverse genetic approach for producing patatin-knockdown potato tubers. Based upon sequence similarity within 29 potato patatin genes available from NCBI, a partial cDNA (635 nts) of *pat3-k1 *was chosen as a target sequence to silence patatin multi-genes of Desiree. Ten independent patatin-hpRNAi lines were generated. In order to test the effect of gene silencing of patatins, the total soluble protein extracted from patatin hpRNAi lines was analyzed on 1D SDS-PAGE gel. Patatin bands were detected in line 2 but not in nine other hpRNAi lines (See Figure 3). Since patatins account for ~40% of total soluble protein in potato tubers [2], the highly reduced patatin content provided an easy measurement of the degree of gene silencing in the individual hpRNAi lines. The observed suppression of patatins in patatin hpRNAi lines was analyzed in more detail using two-dimensional (2D) gel electrophoresis. 2D gel of WT plants showed typically thick/various patatin spots of 40 to 45 kDa, whereas major protein spots were not observed in the gel of hpRNAi line 4 (See Additional file 1). Based upon the measurement of the relative patatin content between the hpRNAi line 4 and the WT plant, patatins in line 4 were reduced by about 99%. The results of 2D gel analysis suggested that individual genes of the patatin multi-gene family were significantly knocked down by RNA interference. A study of patatin antisense transgenic potato plants showed that the amount of patatin proteins in the plants is maximally reduced by 75–90% [28]. Our 2D gel analysis showed that patatin hpRNAi suppressed up to 99% of potato patatins. The data demonstrated that the RNAi approach seems to be much more effective than the antisense RNA approach. In addition to the significant reduction of potato patatins, the level of patatin transcripts was examined by a quantitative real-time RT-PCR. The levels of patatin transcripts were dependent on the lines. The maximum reduction of 99% in the transcript level of *pat3-k1 *was observed in line 4, in comparison with that of the control PCR (See Figure 4). The decreased levels of transcripts in patatin-hpRNAi line 4 were significantly consistent with the suppression of patatins (See Additional file 1C). Since primers of real-time RT-PCR were designed at the highly conserved gene (*pat3-k1*), the dramatic reduction in *pat3-k1 *transcripts probably includes the knockdown of other patatin genes as well. Furthermore, based upon the fact that various patatin spots were not observed in the 2D gel of patatin hpRNAi line 4, the transcripts of most patatin genes would be effectively degraded by RNA interference. Overall, the maximum level of reduction in patatin RNAs and proteins in the hpRNAi lines was approximately 99%.

Human-like glycoproteins have been produced in plant systems, including potato and tobacco [29-32]. Use of more advantageous protein expression systems is necessary for plant-based human-like glycoprotein production [reviewed in [33]] because the yield of recombinant proteins is very low and because expensive purification steps are required. Patatin knockdown potatoes could be used as a suitable expression host for the production of human-like glycoproteins, since very low endogenous glycoprotein patatins might make a relatively selective purification of human-like glycoproteins easier, with less patatin contamination (See Figure 5). Furthermore, the use of patatin tuber specific promoter and N-terminal targeting sequences could easily drive the expression of human glycoproteins in the vacuoles of patatin knockdown potato tubers.

Glycosylation, the attachment of oligosaccharides to proteins, is one of the major posttranslational modifications of proteins in plants and animals. In most therapeutic glycoproteins, the sugar is attached to the Asn residue of a tripeptide Asn-X-Ser/Thr of the protein and/or Thr or Ser residues in the peptide backbone [reviewed in [33]]. The processing of the N-linked glycans occurs in ER and Golgi apparatus. Plant and mammalian N-glycan maturations differ only in the late Golgi apparatus [reviewed in [33]]. In terms of the production of human therapeutic glycoproteins in plant systems, it is necessary to inhibit undesirable plant-specific posttranslational modifications in plants and to glycosylate target proteins in a manner similar to human systems via genetic engineering [reviewed in [34,35]]. Therefore, the first prerequisite for the production of human therapeutic glycoproteins in potato tubers is to suppress the expression of major glycoprotein "patatins". Thus, the patatin-knockdown potato tuber would be a practical candidate to host the selective production of human therapeutic glycoproteins.

## Conclusion

RNA silencing technology was used to produce patatin-knockdown potato tubers. A target sequence (635 nts) of highly conserved gene (*pat3-k1*) in the patatin multi-gene family was amplified for the construction of a patatin-specific hairpin RNAi vector. The effects of gene silencing in patatin-hpRNAi lines were analyzed at the protein level by both 1D and 2D PAGE gel electrophoresis. In addition, the gene silencing effects were confirmed at the mRNA level by a quantitative real-time RT-PCR. Dependent on the patatin hpRNAi line, almost all of the individual patatin variants (40~45 kDa) were reduced by approximately 99% at both the protein and mRNA levels in line 4. Despite a significant reduction of patatin glycoproteins in potato tubers, patatin hpRNAi lines showed a normal phenotype during tissue culture and greenhouse cultivation. Furthermore, patatin-knockdown potato tubers allowed rapid purification of other glycoproteins without contamination of patatin. Therefore, patatin-knockdown potato tubers appear to have strong potential as a plant host for the production of human therapeutic glycoproteins.

## Methods

### Plant materials and growth conditions

Potatoes (*Solanum tuberosum *L. cv. Desiree) were cultivated in plate on Murashige and Skoog medium [36] (pH 5.8) containing MS salt, 30 g/l sucrose, Staba vitamin [37], 100 mg/l inositol and 8 g/l agar, which were kept at 24 ± 2°C under light for 16 hr and dark for 8 hr. Tubers were induced with 90 g/l sucrose at 20 ± 2°C under dark conditions. Potato plants were also grown in a greenhouse (22°C during the day and 20°C at night).

### Patatin hpRNAi vector construction

*pat3-k1 *gene [GenBank: DQ114421] was amplified by PCR with Desiree (WT) as a template and with forward and reverse primers (5'-**ATC GAT GGT ACC **CCA TTT AGC TGC CTC TTC TGC TG-3' and 5'-**TCT AGA CTC GAG **GAA CAG GTA CAG GAG GTT TAT TGA C-3'). The primers contained the restriction sites for *Cla*I – *Kpn*I or *Xho*I – *Xba*I to facilitate subcloning, respectively. The amplified cDNA fragment (635 bp) was subcloned into a pKANNIBAL vector [21] predigested with the restriction enzymes as *Xho*I – *Kpn*I or *Cla*I – *Xba*I, respectively. The recombinant pKANNIBAL vector was confirmed by restriction analysis with *Xho*I – *Kpn*I or *Cla*I – *Xba*I digestion and nucleotide sequencing with the synthetic oligonucleotide primers (5'-TAC GTC AGT GGA GAT GTC ACA TC-3' and 5'-ACA TCT CCA CTG ACG TAA GG-3'). pKANNIBAL vector was digested with *Not *I enzyme. *Not *I fragments were cloned into pART27 binary vector [21] and then patatin hpRNAi binary vector was transformed into potato plants (cv. Desiree) via *Agrobacterium*-mediated transformation.

### Transformation of potato plants and selection of transgenic potato plants

Leaves (3–10 mm) from 3 to 4 week old shoots were cut at the base. Three hundred micrograms of bacterial suspension were pelleted and resuspended in 20 ml liquid YEP medium containing 50 mg/l of kanamycin. The bacteria were subsequently cultured at 28°C at 180 rpm in the dark (until OD_600 _reached 0.6). Stems of rejuvenated shoots were cut into 0.4 to 0.8 cm long segments, immersed on the activated *Agrobacterium *suspension for 10 min, blotted dry on sterile filter paper, and co-cultured for 2 days on the co-culture (CC) medium at light conditions between 300 to 400 μmol photons m^-2 ^sec^-1^. After 2 days, the leaves were placed upside down on PR (plant regeneration) medium containing 0.01 mg/l NAA, 0.1 mg/l GA_3_, 2 mg/l Zeatin, 100 mg/l kanamycin, 500 mg/l carbenicillin for the selection of stable transformants. Every 2 weeks, leaves with callus were transferred to new PR medium. After 7 to 8 weeks, the first shoots that grew to 0.5 cm in height were transferred to a new medium containing 100 mg/l carbenicillin and 50 mg/l kanamycin. All shoots were harvested within a period of 10 weeks. After 1 to 2 weeks, the regenerated shoots were cultured in MS medium. The transgene in the hpRNAi lines was confirmed by PCR screening with *npt *II primers (5'-ATG ATT GAA CAA GAT GGA TTG CAC-3', 5'-TCA GAA GAA CTC GTC AAG AAG GCG-3') and CaMV 35S primers (5'-TAC ACA ACA AGT CAG CAA ACA GAC-3', 5'-TAC GTC AGT GGA GAT GTC ACA TC-3').

### Southern blot analysis

The genomic DNA was digested with *Xba *I to confirm the copy number of inserted T-DNAs for the patatin-hpRNAi derived transformants. The digested DNA was electrophoresed on 1% agarose gel and then transferred to positively charged nylon membrane (Roche Co., Germany) using a Turboblotter system (Schleicher & Schuell). Dig-labeled probes were generated by PCR Dig Labeling Mix (Roche Co., Germany) with *npt *II primer (5'-ATG ATT GAA CAA GAT GGA TTG CAC-3', 5'-TCA GAA GAA CTC GTC AAG AAG GCG-3'). DNA gel blots were hybridized at 42°C in a DIG easy hybridization buffer (Roche Co., Germany). After hybridization overnight, the membrane was washed as follows: 2 × SSC, 0.1% SDS, 58°C; 0.5 × SSC, 0.1% SDS, 58°C. Each washing step was performed twice for 5 min. The membrane was then detected using the Dig Detection Kit following the manufacturer's instructions (Roche Co., Germany).

### SDS-PAGE analysis

SDS-PAGE was performed with 10% (w/v) acrylamide gels according to Sambrook and Russell (2001). Molecular weight protein marker PageRuler™ Prestained Protein Ladder (Fermentas) was run simultaneously for each gel. The protein bands were visualized by coomassie blue staining.

### Two-Dimensional Electrophoresis

Total protein extracts were prepared from potato micro-tubers (20 days since initiation of micro-tubers) of the hpRNAi lines and WT plants. Tubers were ground using a mortar and pestle in liquid nitrogen. Total proteins were extracted at 4°C in 1 ml of thiourea/urea lysis buffer containing 8 M urea (Sigma), 4% (w/v) CHAPS (Amersham Pharmacia Biotech), carrier ampholytes (Amersham Pharmacia Biotech), 40 mM Trizma base (Sigma), and the protease inhibitor cocktail "complete EDTA-free" from Roche Diagnostics (Mannheim, Germany). Protein concentrations of the total protein extract were measured according to Bradford [38]. Bovine serum albumin was used as a standard. 200 μg protein extracts were separated using gel strips forming an immobilized nonlinear pH gradient from 4 to 7 (Immobiline DryStrip, pH 4–7 NL, 17 cm; Bio-Rad Protein IEF Cell). Strips were rehydrated for 12 h at 22°C with the thiourea/urea lysis buffer and the protein extracts. Isoelectrofocusing was performed at 20°C in the Multiphor II system (Bio-Rad Protein IEF Cell) and isoelectrofocusing of 17 cm pH 4–7 L IPG strips (Bio-Rad) were formed using an IPGPhor first dimension electrophoresis unit (Bio-Rad) for 12 hr at 50 V, 15 min 250 V, 3 hr at 250 V, and 6 hr gradient to 10,000 V. Prior to the second dimension, the gel strips were equilibrated for 2 × 20 min in 2 × 10 ml of equilibration solution containing 6 M urea, 20% (v/v) glycerol, 2% (w/v) SDS, and 1.5 M Tris-HCl (pH 8.8). DTT (130 mM) was added to the first equilibration solution, and iodoacetamide (2.5% [w/v]) was added to the second. Silver staining of gels was performed as described by Heukeshoven and Dernick [39].

### Real-time RT-PCR

Total RNA was extracted from two month old mature potato tubers of ten independent patatin-hpRNAi lines grown in greenhouse using the phenol/SDS method [40]. First-strand cDNAs of *pat3-k1 *were synthesized at 42°C for 15 min by MuLV reverse transcriptase (Applied Biosystems, USA) followed by real-time PCR amplification of patatin-fragments (652 bp) using cDNA-specific primers (5'-ATC AAG CTT TGA CAG ACG TAA GG-3' and 5'-ATA GGT TTC ATG ATT GTC TTC G-3'). Real-time PCR was performed using the Greenstar PCR master mix (Bioneer) on a machine (Bio-Rad, Hercules, CA) according to the manufacturer's protocol. As an internal control for the correct PCR conditions and RNA amount in each sample, cDNA fragments of the constitutively expressed 18S rRNA [GenBank: X62738] were amplified using the following primers: (5'-GGGCATTCGTA TTTCATAGTCAGAG-3' and 5'-CGGTTCTTGATTAATGAAAACATCCT-3'). Results were expressed as fold change relative to WT.

### Purification of glycoprotein on concanavalin A (ConA)-Sepharose

To purify glycoproteins by affinity chromatography, the total soluble protein extracted from potato tubers (two month old) was passed through a ConA-Sepharose 4B (GE healthcare, Freiburg, Germany) column at a flow rate of 12 ml/h. After washing of the matrix, the bound proteins were eluted with 0.4 M methyl-α-D-mannopyranoside in 50 mM Tris/HCl, pH 7.4. The flowthrough and bound proteins were collected and concentrated by methanol precipitation. The glycoproteins were examined by SDS-PAGE analysis.

## Authors' contributions

YK carried out the molecular cloning of RNAi vector, SDS-PAGE, transformant analysis, and participated in preparing the manuscript. YL carried out the Real Time PCR analysis and *in vitro *tuberization, and participated in preparing the manuscript. HK carried out the transformation and selection of potato plants. MK carried out the 2-D gel analysis of patatin protein. KH and JJ conceived of parts of the work and helped draft the manuscript. JK carried out the ConA purification of potato glycoprotein. JJ conceived of the study, and participated in its design and coordination and helped draft the manuscript. All authors read and approved the final manuscript. YK and YL contributed equally to this work.

## Supplementary Material

Additional file 1 **2D gel of potato (cv. Desiree) tuber proteins in patatin hpRNAi and WT plants**. (A) 2D gel PI 4–7 of potato tuber protein of WT plants. (B) Patatin spots boxed in gel (A). (C) 2D gel PI 4–7 (the same area as (B)) of potato tuber proteins of patatin hpRNAi line 4. Patatin variants were almost completely suppressed in line 4, when compared to WT plants. Protein was extracted from potato tuber. 200 μg of protein extracts was separated by isoelectric focusing IPG pH 4–7 in the first dimension, and by 12.5% SDS/PAGE in the second dimension. The proteins were stained with silver nitrate. Click here for file

Additional file 2 ***In vitro *and *ex vitro *grown patatin hpRNAi lines display normal growth phenotype**. (A) Wild-type and patatin hpRNAi lines grown *in vitro*. *In vitro *grown potato plants were cultivated at 24 ± 2°C for 4 weeks and then tuberization was induced at 20 ± 2°C for 4 weeks. WT and patatin hpRNAi lines produced round pink micro-tubers in various sizes *in vitro*. (B) Potato tubers of patatin hpRNAi lines and WT plants grown in a greenhouse for 14 weeks. (C) The number of potato tubers per plant and average tuber weight in WT and patatin hpRNAi lines grown in greenhouse. Lanes 1 to 10 are patatin hpRNAi potato lines. Mean and SD were derived by measurement of five plants per line. The error bars indicate mean ± SD. There were no significant difference in the mean values between WT and patatin hpRNAi lines, when assessed with Anova's *t*-test at *P *< 0.01, respectively. (D) Patatin hpRNAi lines and WT plants grown in greenhouse for 8 weeks. There were no phenotypic differences between WT and patatin hpRNAi lines. Click here for file
