# Supplementary figures and images for: Development of patatin knockdown potato tubers using RNA interference (RNAi) technology, for the production of human-therapeutic glycoproteins
Source: BMC Biotechnol. 2008 Apr 3;8:36. doi: 10.1186/1472-6750-8-36 (PMC2335101; doi:10.1186/1472-6750-8-36)

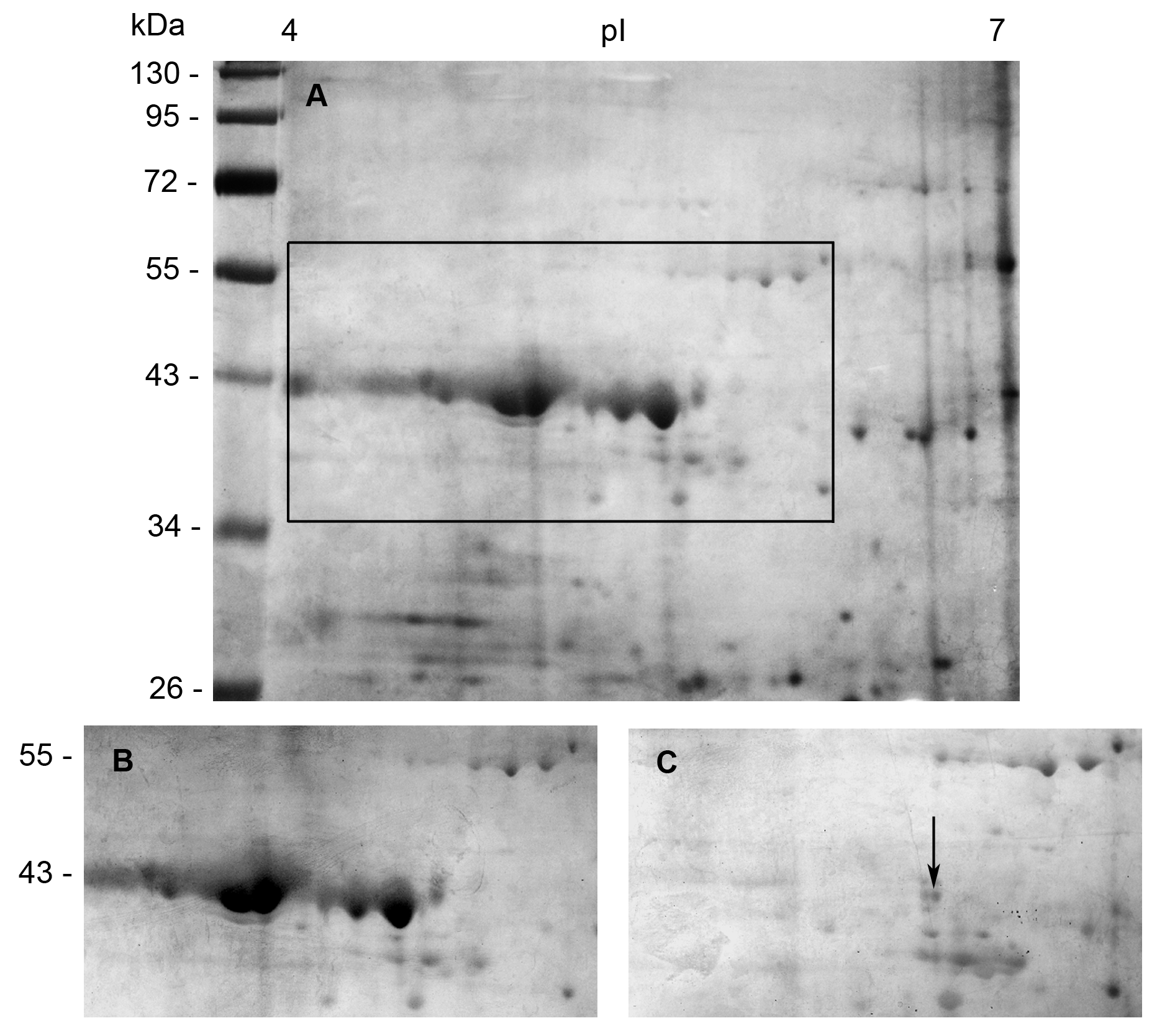

Supplement: Additional file 1 — 2D gel of potato (cv. Desiree) tuber proteins in patatin hpRNAi and WT plants. (A) 2D gel PI 4–7 of potato tuber protein of WT plants. (B) Patatin spots boxed in gel (A). (C) 2D gel PI 4–7 (the same area as (B)) of potato tuber proteins of patatin hpRNAi line 4. Patatin variants were almost completely suppressed in line 4, when compared to WT plants. Protein was extracted from potato tuber. 200 μg of protein extracts was separated by isoelectric focusing IPG pH 4–7 in the first dimension, and by 12.5% SDS/PAGE in the second dimension. The proteins were stained with silver nitrate. [file 1472-6750-8-36-S1.tiff]

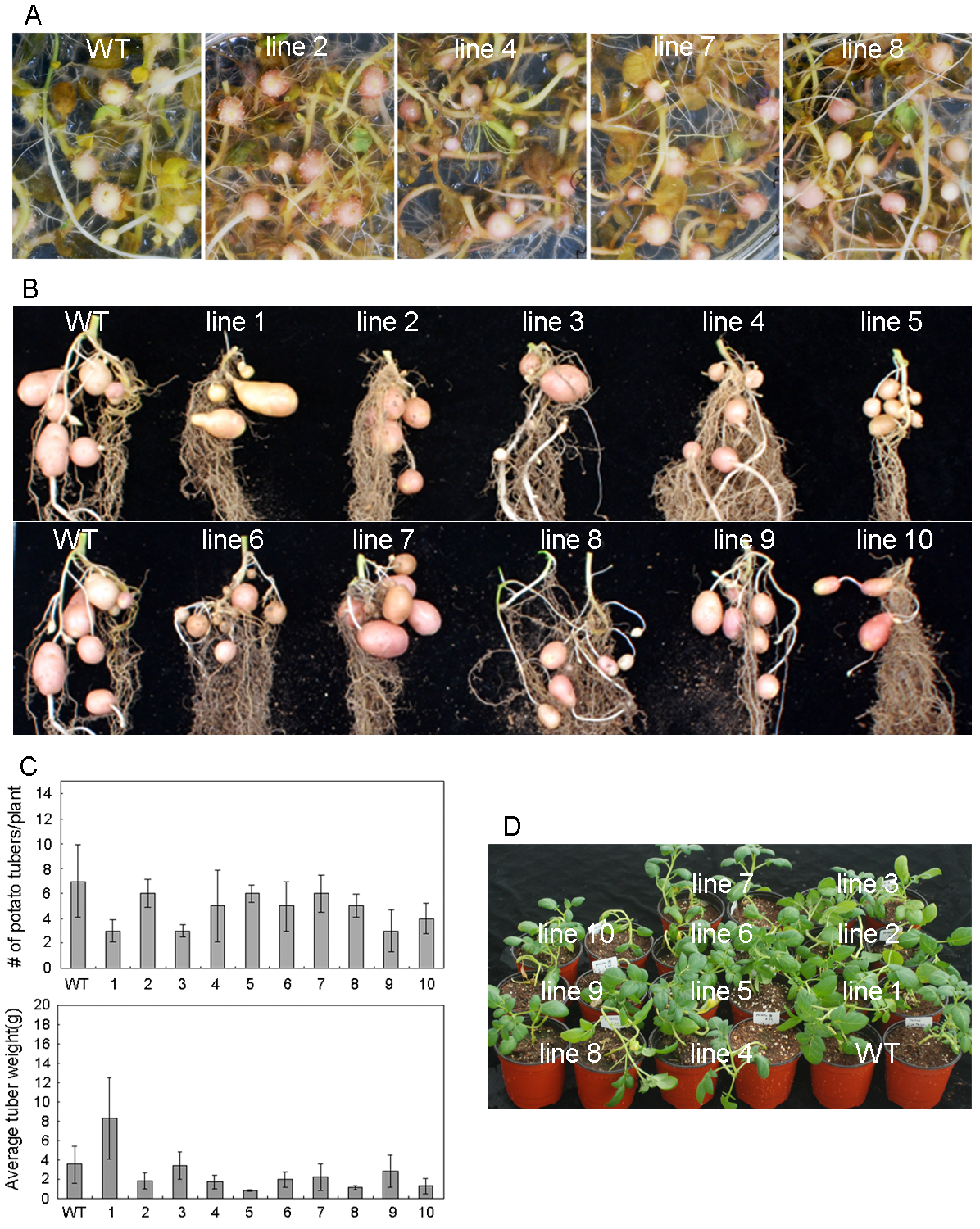

Supplement: Additional file 2 — In vitro and ex vitro grown patatin hpRNAi lines display normal growth phenotype. (A) Wild-type and patatin hpRNAi lines grown in vitro. In vitro grown potato plants were cultivated at 24 ± 2°C for 4 weeks and then tuberization was induced at 20 ± 2°C for 4 weeks. WT and patatin hpRNAi lines produced round pink micro-tubers in various sizes in vitro. (B) Potato tubers of patatin hpRNAi lines and WT plants grown in a greenhouse for 14 weeks. (C) The number of potato tubers per plant and average tuber weight in WT and patatin hpRNAi lines grown in greenhouse. Lanes 1 to 10 are patatin hpRNAi potato lines. Mean and SD were derived by measurement of five plants per line. The error bars indicate mean ± SD. There were no significant difference in the mean values between WT and patatin hpRNAi lines, when assessed with Anova's t-test at P < 0.01, respectively. (D) Patatin hpRNAi lines and WT plants grown in greenhouse for 8 weeks. There were no phenotypic differences between WT and patatin hpRNAi lines. [file 1472-6750-8-36-S2.tiff]
